# Optimization of Supercritical Carbon Dioxide Extraction of *Saussurea costus* Oil and Its Antimicrobial, Antioxidant, and Anticancer Activities

**DOI:** 10.3390/antiox11101960

**Published:** 2022-09-30

**Authors:** Hanaa Y. Ahmed, Sayed M. Kareem, Ahmed Atef, Nesreen A. Safwat, Reda M. Shehata, Mohammed Yosri, Mahmoud Youssef, Morooj M. Baakdah, Rokayya Sami, Roua S. Baty, Nouf H. Alsubhi, Ghadeer I. Alrefaei, Ali A. Shati, Fahmy G. Elsaid

**Affiliations:** 1 The Regional Center for Mycology and Biotechnology, Al-Azhar University, Nasr City, Cairo 11787, Egypt; 2 Food Science and Technology Department, Faculty of Agriculture, Al-Azhar University, Nasr City, Cairo 11651, Egypt; 3 Department of Chemistry, Preparatory Year Program, Batterjee Medical College, P.O. Box 6231, Jeddah 21442, Saudi Arabia; 4 Department of Food Science and Nutrition, College of Sciences, Taif University, P.O. Box 11099, Taif 21944, Saudi Arabia; 5 Department of Biotechnology, College of Science, Taif University, P.O. Box 11099, Taif 21944, Saudi Arabia; 6 Biological Sciences Department, College of Science and Arts, King Abdulaziz University, P.O. Box 80200, Rabigh 21911, Saudi Arabia; 7 Department of Biology, College of Science, University of Jeddah, P.O. Box 80327, Jeddah 21589, Saudi Arabia; 8 Biology Department, Science College, King Khalid University, P.O. Box 960, Abha 61421, Saudi Arabia; 9 Zoology Department, Faculty of Science, Mansoura University, Mansoura 35516, Egypt

**Keywords:** supercritical fluid extraction, oils, antimicrobial, anticancer, antioxidant

## Abstract

*Saussurea costus* is a medicinal plant with different bioactive compounds that have an essential role in biomedicine applications, especially in Arab nations. However, traditional extraction methods for oils can lead to the loss of some volatile and non-volatile oils. Therefore, this study aimed to optimize the supercritical fluid extraction (SFE) of oils from *S. costus* at pressures (10, 20, and 48 MPa). The results were investigated by GC/MS analysis. MTT, DPPH, and agar diffusion methods assessed the extracted oils’ anticancer, antioxidant, and antimicrobial action. GC/MS results showed that elevated pressure from 10 to 20 and 48 MPa led to the loss of some valuable compounds. In addition, the best IC_50_ values were recorded at 10 MPa on HCT, MCF-7, and HepG-2 cells at about 0.44, 0.46, and 0.74 μg/mL, respectively. In contrast, at 20 MPa, the IC_50_ values were about 2.33, 6.59, and 19.0 μg/mL, respectively, on HCT, MCF-7, and HepG-2 cells, followed by 48 MPa, about 36.02, 59.5, and 96.9 μg/mL. The oil extract at a pressure of 10 MPa contained much more of á-elemene, dihydro-à-ionone, patchoulene, á-maaliene, à-selinene, (-)-spathulenol, cedran-diol, 8S,13, elemol, eremanthin, á-guaiene, eudesmol, ç-gurjunenepoxide-(2), iso-velleral, and propanedioic acid and had a higher antioxidant activity (IC_50_ 14.4 μg/mL) more than the oil extract at 20 and 48 MPa. In addition, the inhibitory activity of all extracts was higher than gentamicin against all tested bacteria. One of the more significant findings from this study is low pressure in SFE enhancement, the extraction of oils from *S. costus,* for the first time. As a result, the SFE is regarded as a good extraction technique since it is both quick and ecologically friendly. Furthermore, SFE at 10 MPa increased the production and quality of oils, with high antioxidant activity and a positive effect on cancer cells and pathogens.

## 1. Introduction

Herbals, also known as medicinal plants, are the natural sources of various medical compounds that are considered promising candidates for controlling and preventing infectious diseases without side effects [1]. Herbs and their extracts have been used in traditional medicine in the ancient age. In the present age, more than 80% of people still depend on herbal medicine because it contains natural medical compounds. For example, but not limited to medical compounds, can inhibit the growth of different pathogenic microbes and can also suppress the growth of different types of cancer cells [2]. Furthermore, herb extracts possess antioxidant properties that play an important role in reducing oxidative stress [3]. Herbs and their extracts are considered safe for usage not only in the pharmaceutical sector but are extended to include cosmetics, food additives, and nutrition sectors [1]. Nowadays, nearly 85,000 medicinal plant species are used throughout the world. However, much more of these plants with innumerable phytochemicals are unstudied entirely; among such medicinal plants is *Sausurea costus*, which belongs to the Asteraceae family [3].

*S. costus* is known as “Al-Kost Al-Hindi” in Arab nations and has been utilized by conventional healers since the dawn of Islamic civilization. Recently, researchers have paid more attention to the therapeutic characteristics of *S. costus,* represented as an antioxidant, antiulcer, anti-inflammatory, anti-immune, stimulant, disinfectant, repellant, sedative, bronchodilator, antibacterial, anticancer, antiviral activity, and so on [4,5,6,7,8]. The therapeutic characteristics of *S. costus* contain nearly 25,000 functional bioactive compounds [9], such as sesquiterpene lactones, flavonoids, phenolics, alantolactone, alkaloids, terpenes, costunolide, dehydrocostus lactone, and essential oils [10]. Extraction of these medicine components is one of the vital methods in obtaining herb medicine of high grade. Conventional methods are commonly used to separate the target compounds in herbs, such as distillation by steam, hydro-distillation, solvent, infrared-assisted extraction, and Soxhlet extraction, due to their ease of use. Several studies have used single and mixed solvents, such as ethanol, n-butanol, chloroform, methanol, ethyl acetate, and n-hexane, to extract medicinal compounds from *S. costus* [11,12,13]. Other studies have also combined solvents with temperatures via the Soxhlet apparatus [14] and infrared-assisted extraction [15] to obtain a high yield of medicinal compounds from *S. costus*. However, all the previously mentioned conventional methods suffer from some serious restrictions implicating the usage of extensive quantities of poisonous solvents, high thermals, length of extraction times, solvent remains in the final product, and the necessity to discard so. Further, the extraction efficiencies of these methods primarily depend on heat and extraction time to promote the dissolution and propagation of solutes. These break down medicine plants’ volatiles, antioxidants, and sensitive bioactive compounds [16].

Extraction methods relating to sensitive bioactive compounds from medicinal plants have been improved using green chemistry extraction methods to vent waste safely. Supercritical fluid extraction (SFE) is an advanced green method with unique selectivity for extracting sensitive bioactive compounds from herbs and medicinal plants, such as essential oils [17]. Reports reveal that this green method has many benefits over conventional extraction methods, such as non-toxic solvents, lower temperatures, short extraction time, and eco-friendly [18,19]. One powerful advantage of using SFE applies engaging carbon dioxide as a solvent with high diffusivity power, cheap, easy to get, non-toxic, and authorized for food and pharmaceutical usage [18,20]. Many previous studies have reported that SEF-CO_2_ extraction method under high pressure enables the extraction of volatile oils and oleoresins from plant materials [21]. Furthermore, SEF-CO_2_ extraction improves the extraction of phenolic, flavonoid, and antioxidant compounds from plant materials [22,23]. Despite the many advantages of using the SEF-CO_2_ extraction method, no studies have been conducted on the extracted bioactive components from *S. costus* using the SEF-CO_2_ extraction method, which encouraged us to do this research. Therefore, the current study was designed to (1) extract high-value compounds from *S. costus* by using the SEF-CO_2_ extraction technique under different pressure conditions, then, (2) separate and identify bioactive compounds by GC/MS, finally, (3) to investigate the antimicrobial, antioxidant, and anticancer activity of those high-value compounds extracted from *S. costus*. 

## 2. Materials and Methods

### 2.1. Collection of Plant Material

*S. costus* powder was obtained from the local market (Kingdom of Saudi Arabia). The powder was stored at room temperature in a plastic container.

### 2.2. Supercritical Carbon Dioxide Extraction of Costus Oil 

Five grams of *S. costus* powder proceed in a supercritical fluid extractor. Carbon dioxide from the cylinder was pushed into the extractor chamber through a high-pressure pump after passing through the chiller. The extraction unit’s control panel was programmed with the working temperature (40 °C), pressure 10 MPa (E10), 20 MPa (E20), and 48 MPa (E48), and extraction duration (30 min). When the necessary pressure and temperature were achieved, the extractor opened the valve between the pump and the sample cartridge, allowing 5mL/min of CO_2_ to pass through the sample. The extracted oil was collected in a glass vial after each extraction.

### 2.3. GC-MS Analysis and Conditions

The extracted oil was analyzed by a Thermos Scientific TRACE 1310 Gas Chromatograph (Waltham, MA, USA) attached to an ISQ LT single quadrupole mass spectrometer equipped with a capillary DB-1 column 15 m × 0.25 mm (J & W Scientific, Folsom, CA, USA). The injection port temperature was maintained at 200 °C, and the column oven temperature program was set from 115 °C (1 min) to 280 °C (3 min) (7.5 °C/min). The carrier gas was helium (1.5 mL/min), and the mass spectra were recorded at 70 eV. The chemical components were identified by comparing their mass fragmentation patterns with those of the standard reference data of the WILEY MASS SPECTRAL DATABASE.

### 2.4. MTT Assay

The extracted oils were tested for cytotoxic effects on four cell lines, namely HepG-2 (Human hepatocellular carcinoma cells), MCF-7 (Breast carcinoma cells), and HCT (colon carcinoma cells). Cells were allowed to adhere for 24 h until confluence, then treated with samples from 500 to 15.63 µg/mL concentration and incubated for 24 h at 37 °C. Then, the new medium was added and treated with 100 µL of MTT solution (5 mg/mL) for 4 h at 37 °C. Absorbance was detected at 570 nm using a microplate reader (SunRise TECAN, Inc., San Jose, CA, USA) [24].

### 2.5. Microscopic Studies

The pictures were acquired by a digital camera coupled with an inverted microscope (CKX41; Olympus, Tokyo, Japan).

### 2.6. Antimicrobial Activity

The antimicrobial activity of the extract at three different pressure levels (10, 20, 48 MPa) was tested against Gram-positive bacteria *B. subtilis* ATCC6633, and *S. aureus* (MRSA) ATCC43300, as well as Gram-negative bacteria *P. aeruginosa* (ATCC27853), *E. coli* (ATCC25922), *K. pneumonia* RCMB005 001 (2), (yeasts) *C. albicans* RCMB 005003(1) ATCC 10231, *C. tropical* RCMB005 004 and filamentous fungi, *A. flavus* (RCMB 002002), *F. oxysporium* RCMB008 001 (2) [25]. 

### 2.7. DPPH Radical Scavenging Activity

A 40 μL of the extract at three different pressure levels (10, 20, 48 MPa) was added to 3 ml of DPPH (0.004% *w*/*v*) methanol solution. Absorbance at 515 nm was measured with a UV-visible spectrophotometer (Milton Roy, Spectronic, El Paso, TX, USA, 1201). The inhibition percent (PI) of the DPPH radical was calculated from the following equation:

PI = [{(*AC* − *AT*)/*AC*} × 100] (1)

where *AC* = Absorbance of the control at t = 0 min and *AT* = absorbance of the sample + DPPH at t = 16 min [26]. Then, the IC_50_ was determined, as described before.

### 2.8. Statistical Analysis

Experiments were carried out three times in total. All data are presented as the mean ± standard deviation (SD).

## 3. Results and Discussion

### 3.1. Effects of Different Pressures of Supercritical CO_2_ Extraction on the Yield of Active Compounds from S. costus

**Figure 1:**
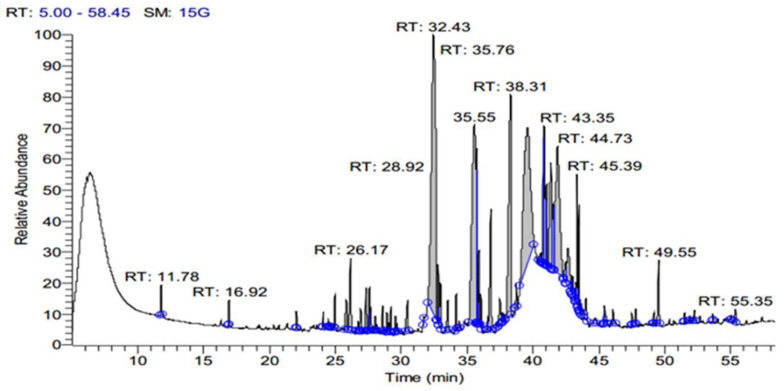
GC-MS Chromatogram of *S. costus* extract by SFE at a pressure of 10 MPa (E10).

**Figure 2:**
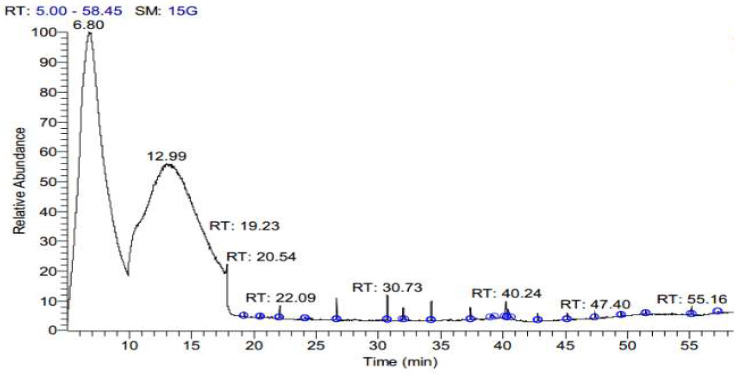
GC-MS Chromatogram of *S. costus* extract by SFE at a pressure of 20 MPa (20).

**Figure 3:**
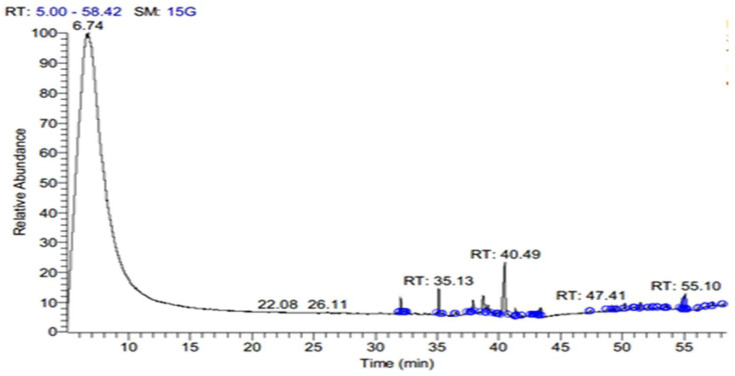
GC-MS Chromatogram of *S. costus* extract by SFE at a pressure of 48 MPa (E48).

The SFE extract of *S. costus* was subjected to GC-MS analysis to determine the bioactive compounds at three different pressure levels (10, 20, 48 MPa). Table 1 shows the effect of pressure on the SFE for extracting the bioactive compound from an *S. costus* at 40 °C and an extraction duration of about 30 min. As shown in Table 1, an increase in pressure caused a decrease in compounds, and different types of compounds occurred. eE10 shows the presence of á-elemene, Dihydro-à-ionone, Patchoulene, á-Maaliene, á-Guaiene, (-)-à-Selinene, (-)-Spathulenol, Cedran-diol, 8S,13, Elemol, Methyl 2-hydroxy-octadeca-9,12,15-trie noate, á-ylangene, gama. -eudesmol, 9,12,15-Octadecatrien-1-ol, (Z, Z, Z), ç-Gurjunenepoxide-(2), Testosterone, Iso-velleral, Furoscrobiculin B, Eremanthin, Chiapin B, Propanedioic acid, Myricanene B, Azuleno[4,5-b] furan-2(3H)-one, Tomentosin. E20 contains different compounds but is low in numbers in comparison with E10, namely, Cyclohexasiloxane, Cycloheptasiloxane, Cyclooctasiloxane, Methyl stearidonate, Cyclononasiloxane, Cyclodecasiloxane, Cholic acid, Lucenin 2, (7,9-Dimethoxy-4-oxo-4H-benzo [d]pyrrolo[3,2,1-ij] quinolone), ((22S,23S,25R)-3-ü-Methoxy-16á,23:22,26-diepoxy-5à-cholestan),(1,3-Bis(4-chlorobenzyl)-5,6-dihydrobenzo[f]quinazoline). Additionally, E48 contains other compounds, Methyl stearidonate, Pleiocarpamine, Gitoxigenin, Effusanin B, (1H-Imidazole,1-[(4-methylphenyl) sulfonyl), Xanthumin, (QUERCETIN 7,3′,4′-TRIMETHOXY), (Card-20(22)-enolide), Prednisone, (25-Norisopropyl-9,19-cyclolanostan-22-en-24-one), (1,4-Pentadien-3-one,1,5-diphenyl), Lucenin 2. The extract contains ISO-VELLERAL, Furoscrobiculin B, and Propanedioic acid, which are also presented at E10 (Figure 1, Figure 2 and Figure 3).

The present study observed that high numbers of compounds were extracted from *S. costus* at a pressure of 10 MPa by supercritical fluid extraction. Moreover, raising the pressure from 10 to 20 and 48 MPa reduced the number of compounds, and other compounds were observed. The change in the number of compounds after extraction under different pressures remains to increase; the pressure causes a solvent density increase; hence, compounds with a larger molecular weight were extracted. The reduction of yield with increasing pressure was also obtained by Machmudah et al. [27] for the total extraction of alkaloids from the leaves of *Ilex paraguariensis*, and Cassel et al. [28] to extract alcohols from the mushroom. Previous research on the principle of SFE showed that increasing the pressure increased the density and diffusivity of the SFE, resulting in enhanced extraction [29]. Higher pressure reduced the mass transfer time, and part of the extracted oil remained in the separator.

In contrast, another study found that volatile oil extraction is unrelated to pressure [29]. At the same time, the compressibility of supercritical CO_2_ is higher at low pressure and decreases at high pressure. Thus, the higher solvating of SC-CO_2_ decreased the extraction selectivity and increased the coextraction of nonvolatile components [30]. Similar observations were reported by Hamburger et al. [31], who found that some nonvolatile lipophilic compounds were coextracted at increased pressure.

### 3.2. Anticancer Activity

**Figure 4:**
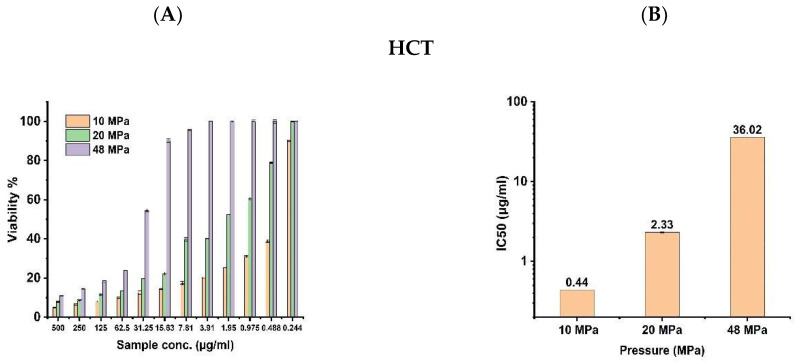

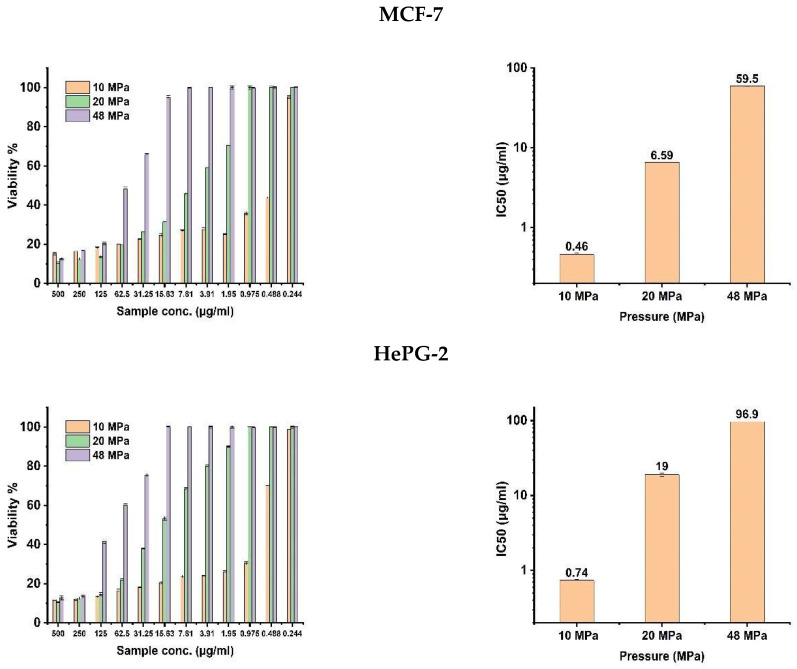
The antitumor activity of SFE extracts of *S. costus* at different pressures after 24 h of treatment against HCT, MCF-7, and HepG-2 cells (mean ± s. d, n = 3). (**A**). Viability %. (**B**). IC_50_ value.

The effects of different pressures of the extractor on the antitumor activity of *S. costus* extract against various cancer cells, namely MCF 7 (Breast carcinoma cells), HCT (colon carcinoma cells), and HepG-2 (liver carcinoma cells), were examined. The three pressure levels were used at 10, 20, and 48 MPa at 40 °C of temperature. It can be seen that there are different effects of the three extracts on anticancer activity. E10 shows highest anticancer activity toward the three tumor cell lines, followed by E20 and E48 (Figure 4A).

The samples were also compared using the IC_50_ values representing the sample efficiency as antitumor agents (Figure 4B). The best IC_50_ values were recorded at E10 of about (0.44, 0.46, and 0.74) μg/mL against HCT, MCF-7, and HepG-2 cells, respectively. This was followed by 20 MPa with IC_50_ (2.33, 6.59and 19.0) μg/mL, respectively, and IC_50_ values at 48 Mpa about (36.02, 59.5, and 96.9) μg/mL. E20 was recorded at about (2.33, 6.59, and 19.0) μg/mL, followed by E48 at about 36.02, 59.5, and 96.9 μg/mL, respectively.

From the results of GC/MS, it was clear that E10 contains a high number of compounds; the SFE system could prepare enough volatile oil for anticancer activity [32,33,34]. Among these compounds are, á-Guaiene, (-)-à-Selinene, (-)-Spathulenol, Cedran-diol, 8S,13, Elemol, Methyl 2-hydroxy-octadeca-9,12,15-trie noate, á-ylangene, gama. -eudesmol, 9,12,15-Octadecatrien-1-ol, (Z,Z,Z), ç-Gurjunenepoxide-(2), Testosterone, Iso-velleral, Furoscrobiculin B, Eremanthin, Chiapin B, Propanedioic acid, Myricanene B, Azuleno[4,5-b]furan-2(3H)-one, Tomentosin. Elemene, a sesquiterpene, is characterized by its anticancer activity against different cell lines. Jiang et al. [34] have reported that β-elemene causes an apoptotic trigger on cancer cells. (Dihydro-à-ionone), β-Ionone is an end-ring analog of β-carotenoids, with anti-metastatic properties in vitro and in vivo [35]. Eremanthin is a volatile oil that belongs to the guaianolides and derivatives class of compounds. The eremanthin extracted from *Costus speciosus* was found to stop the proliferation of MCF-7 and MDA-MB-231; it was found that eremanthin regulates cell growth by changing the expression of different signaling molecules [36]. Spathulenol is a tricyclic sesquiterpenoid compound with high anticancer activity [37].

### 3.3. Morphological Studies

**Figure 5:**
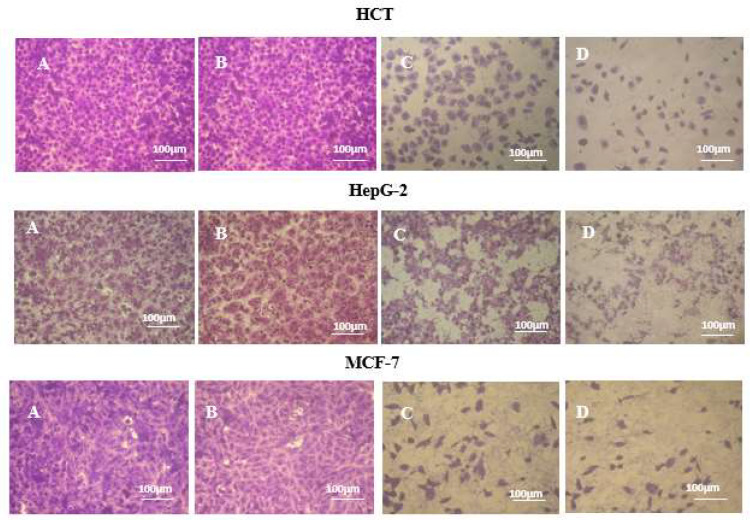
Morphological characteristics of HCT, MCF-7, and HepG-2 cell lines after 24 h of treatment with (15.63 µg mL^−1^) of different extracts. The cancer cells were stained with a crystal violet stain. (**A**) Control cells at 24 h, (**B**) E48, (**C**) E20 MPa, (**D**) E10 Magnification: ×20.

Figure 5 depicts the alterations in the morphology of the HCT, MCF7, and HepG-2 cell lines after treatment with SFE extracts at various pressures. The images taken with an inverted microscope detected a change in the treated cells’ morphology. The control cells had adherent development and a polygonal form, as seen in panel (A) in these images. Inverted microscopy indicated substantial alterations in HCT, MCF-7, and HepG-2 cells following incubation with 15.63 µg/mL of E10, followed by E20 and E48 (Figure 5B–D). *S. costus* contains different bioactive compounds, such as costunolide, sesquiterpene lactones, dehydrocostus lactone, and cynaropicrin, which have antitumor activities for different types of cancer, such as leukemia [38,39], HepG-2 cancer [40], and MCF-7 cancer [41,42].

### 3.4. Antimicrobial Activity

The antimicrobial activity of the extracted oils was measured versus Gram-positive and Gram-negative bacteria, yeasts, and filamentous fungi at different pressures. The antimicrobial activity was done by well diffusion assay at 50 mg/well; inhibition zones were measured in mm and are shown in Table 2. The SFE extract exhibited higher inhibitory activity than the standard antibiotic gentamicin. Moreover, higher inhibition was observed at E10, followed by E20 and E48. The inhibition zones of the SFE extract at E10 against *S. aureus* (MRSA), *B. subtilis*, *E. coli*, *P. aeruginosa,* and *K. pneumonia* were about 52, 50, 40, 42, and 50 mm, respectively. In contrast, the inhibition zone at E20 was measured as 41, 46, 35, 30, and 50 mm, respectively. Additionally, E48 was estimated to be about 40, 45, 32, 28, and 49 mm, respectively. At the same time, high inhibitory activity was observed against *C. albicans* and *C. tropicalis* but was still less potent than the reference compound used in this study. The inhibition zones of the SFE extract at E10, E20, and E48 were about 18, 17, and 17 mm, respectively, against *C. albicans* and 15, 12, and 12 mm against *C. tropicalis*. In addition, no inhibitory activity was observed against *A. flavus* or *F. oxysporium* in the three extracts. Different studies have been reported on extracting non-antibiotic substances from natural sources with potential antimicrobial effects in treating multidrug-resistant bacteria, such as SFE extracts [43].

Higher inhibitory effects were observed against *E. coli*, *B. cereus*, *L. monocytogenes*, *S. typhimurium*, and *P. fluorescens* [44] after treatment with the volatile compound of marjoram (*Origanum majorana*) and oregano extracted by SFE. Moreover, the inhibitory activity of rosemary SFE extract has been observed against *S. aureus* and *B. subtilis*. The SFE extracts of *Cinnamomum cassiabuds* showed high antimicrobial activity [45]. Furthermore, the SC extract of *R. cinnamomi* showed better antimicrobial activity than ethanol extraction [46]. The volatile compounds of *Phyllanthus emblica* extracted by SFE showed high activity against *S. aureus*, *B. subtilis*, and *B. cereus* [47]. Costus essential oil affected *Acinetobacter* spp., *E. coli*, *s. aureus*, *P. aeruginosa*, and *Proteus* spp. more than Caraway essential oil. Reddy and Jose found a high inhibition zone using *Costus pictus* leaf oil against various bacterial isolates compared with multiple antibiotics. Similarly, Majumdar and Parihar showed high antimicrobial effects against different bacterial strains. The action of volatile oils on bacterial cells is related to the oil’s effect on the external membrane and cytoplasm [48].

### 3.5. Antioxidant Activity

**Figure 6:**
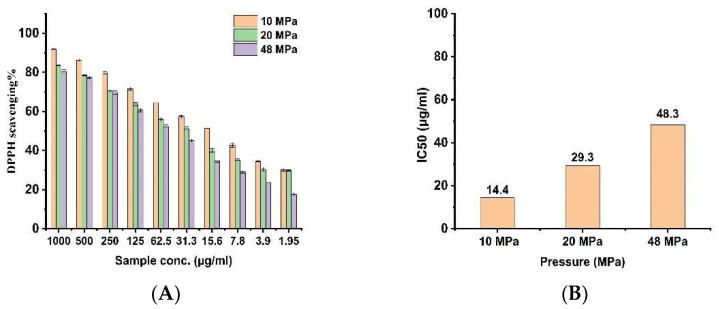
Antioxidant activity of SFE extracts of *S. costus* at different pressures using a DPPH assay (mean ± s. d, n = 3). (**A**). DPPH scavenging %. (**B**). IC50 value.

The antioxidant activities of the SFE extract *S costus* at different pressures were determined using the DPPH method (Figure 6). The results (Figure 6B) are expressed as a 50% reduction in the free radical scavenging concentration in the sample (IC_50_). The extract at E10 demonstrated the greatest activity (IC_50_ of 14.4 μg/mL) among the extracts obtained at various pressures. The IC50 values of the extract was 29.3 μg/mL and about 48.3 μg/mL with E20 and E48, respectively.

The highest antioxidant remains in the different oils in the costus plant, such as á-elemene, Dihydro-à-ionone, Patchoulene, á-Maaliene, à-Selinene, (-)-Spathulenol, Cedran-diol, 8S,13, Elemol, Eremanthin, á-Guaiene, eudesmol, ç-Gurjunenepoxide-(2), Iso-velleral, and Propanedioic acid. The results are consistent with those obtained by [49], which indicated that the ethanolic extract of *S. costus* contains different oils with better antioxidant activity of IC_50_ = 0.12325 mg/mL among these oils (+)-Isovalencen, Valerenol, Eudesm-4(14)-en-11-ol, Trans-á-Ionone, Beta-Guaiene, Gamma-guarjumenepoxide-(2), 1,3-propanediol,2-(hydroxymethyl)-2-nitro-, and Farnesene epoxide. In addition, the high antioxidant activity of methanolic extract of costus roots and seeds can be related to the high amount of phenols and flavonoids [50,51].

The highest activity of oil can be explained by selecting the best extraction method, such as SFE, and optimizing different parameters, such as pressure, that can produce high-yield extracts with minimum modifications to the functional characteristics of the extract. Plants often have modest amounts of physiologically active compounds [52]. Essential oils are complex mixes containing a wide range of components. Many researchers on the antioxidant activities of essential oils include terms like synergism, antagonism, and additivity [53]. Furthermore, the essential oil components’ strong reducing power showed that they could operate as electron donors and reduce the oxidized intermediate of lipid peroxidation, allowing them to act as antioxidants [53].

## 4. Conclusions

This is the first study using the SFE technique to extract a high yield of oils from *S. costus*. The extracted oil’s yield and quality have been optimized by modifying the pressure of the SFE apparatus. Moreover, the GC-MS method has identified nearly 70 bioactive compounds involving a high number of oils in extracted *S. costus*, which are known to have potent antioxidant activity in addition to their therapeutic effects. Interestingly, the extracted yield increased at low pressure (10 MPa) compared to the other treatment conditions. All *S. costus* extracts showed high antioxidant, anticancer, and antimicrobial activities; nevertheless, the highest antioxidant, anticancer, and antimicrobial activities were observed at 10 MPa compared to the other two pressures used. Finally, the insights gained from this study may assist in applying green chemistry to extract high-value compounds with high therapeutic effects from medicinal plants. Further research needs to be conducted on animal models to verify the therapeutic effects of the *S. costus* extracts obtained in this study.

## Figures and Tables

**Table 1:** Chemical constituents identified from the *S. costus* extract by SFE at different pressures.

Chemical Name	10 MPa		20 MPa		48 MPa	
	Peak Area (%)	RT	Peak Area (%)	RT	Peak Area (%)	RT
á-elemene	0.40	24.79	-	-	-	-
Dihydro-à-ionone	0.64	25.84	-	-	-	-
à-Ionone	1.56	26.17	-	-	-	-
Patchoulene	0.41	26.92	-	-	-	-
á-Maaliene	0.69	27.36	-	-	-	-
á-Guaiene	0.40	27.70	-	-	-	-
(-)-à-Selinene	0.15	28.02	-	-	-	-
(-)-Spathulenol	0.09	28.13	-	-	-	-
Cedran-diol, 8S,13	0.33	28.60	-	-	-	-
á-Maaliene	0.22	28.92	-	-	-	-
á-Guaiene	0.4	29.07	-	-	-	-
Elemol	0.23	29.56	-	-	-	-
Methyl2-hydroxy-octadeca-9,12,15-trienoate	0.11	29.66	-	-	-	-
á-ylangene	0.71	30.50	-	-	-	-
gama-eudesmol	0.08	31.69	-	-	-	-
9,12,15-Octadecatrien-1-ol,(Z, Z, Z)	18.61	32.43	-	-	-	-
ç-Gurjunenepoxide-(2)	2.06	32.79	-	-	-	-
ç-Gurjunenepoxide-(2)	0.71	32.98		-	-	-
ç-Gurjunenepoxide-(2)	0.56	33.53		-	-	-
12-Oxatetracyclo[4.3.1.1(2,5).1 (4,10)]dodecane,11-isopropylidene-	0.49	34.17	-	-	-	-
Bicyclo[4.4.0]dec-2-ene-4-ol,2-methyl-9-(prop-1-en-3-ol-2-yl)-	11.85	35.55	-	-	-	-
Methyl 8,10-octadecadiynoate	2.42	35.76	-	-	-	-
Begonanline	2.89	36.82	-	-	-	-
ISO-VELLERAL	0.09	37.25	-	-	-	-
Hexadecanoic acid, methylester (CAS)	8.25	38.31	-	-	-	-
Testosterone	0.29	38.73	-	-	-	-
Eremanthin	14.71	39.57		-		-
ISO-VELLERAL	1.40	40.78	-	-	0.27	37.71
Methyl4,7,10,13,16-docosapentaenoate	3.10	40.89		-	-	-
Furoscrobiculin B	1.33	41.04	-	-	-	-
Eremanthin	0.24	42.95	-	-	-	-
Eremanthin	0.36	43.02	-	-	-	-
Eremanthin	0.19	43.23	-	-	-	-
Chiapin B	1.09	43.35	-	-	-	-
Azuleno[4,5-b]furan-2(3H)-one,	1.18	43.49	-	-	-	-
Furoscrobiculin B	0.09	43.56	-	-	-	-
Carda-5,20(22)-dienolide, 3,14,19-trihydroxy-, (3á)-(CAS)	0.18	44.73	-	-	-	-
Cyclodecasiloxane,eicosamethyl-	0.20	45.39	-	-	-	-
Myricanene B	0.21	47.80	-	-	-	-
Di-(2-ethylhexyl)phthalate	1.10	49.55	-	-	-	-
Eremanthin	-		-	-	5.41	39.10
Eremanthin	-	-	-	-	33.87	40.49
Chiapin B	-			-		
Furoscrobiculin B	-		-	-	0.27	39.78
Furoscrobiculin B	-	-	-	-	0.05	39.85
Furoscrobiculin B	-	-	-	-	0.10	39.92
Furoscrobiculin B	-	-	-	-	0.04	39.98
1,3-Bis(4-chlorobenzyl)-5,6-dihydrobenzo[f]quinazoline	-		6.23	22.09	-	
Cycloheptasiloxane	-		10.33	26.64	-	
Cyclooctasiloxane	-		11.81	30.73	-	
Methylstearidonate	-		10.01	32.01	4.04	32.04
Cyclononasiloxane	-		8.74	34.25	-	
Cyclodecasiloxane	-		5.75	37.38	-	
Cholic acid	-		15.14	40.33	-	
Pleiocarpamine	-		-		0.02	
Gitoxigenin	-		-		0.05	32.29
Effusanin B	-		-		0.08	23.39
1H-Imidazole,1-[(4-methylphenyl)sulfonyl]-	-		-		0.38	32.55
Xanthumin	-		-		6.87	35.13
QUERCETIN7,3′,4′-TRIMETHOXY	-		-		3.36	36.49
Card-20(22)-enolide	-		-		0.87	37.56
Prednisone	-		-		0.01	41.90
Lucenin 2	0.26	55.35	3.79	39.12	0.65	42.81
25-Norisopropyl-9,19-cyclolanostan-22-en-24-one,	-		-		0.09	43.01
Propanedioic acid	-				0.11	46.35
1,4-Pentadien-3-one,1,5-diphenyl-	-		-		0.01	48.71

**Table 2:** Antimicrobial activities of *S. costus* extract by SFE at different pressures.

Tested Organisms	Inhibitory Activity against the Tested Organism (Zone of Inhibition in mm)			
	10 MPa	20 MPa	48 MPa	St.
Gram-positive bacteria				Gentamycin
*S. Saureus* (MRSA)—ATCC43300	52 ± 0.25	41 ± 0.42	40 ± 0.74	15 ± 0.60
*B. subtilis* ATCC6633	50 ± 0.58	46 ± 0.79	45 ± 0.96	26 ± 0.52
Gram-negative bacteria				Gentamicin
*E. coli* ATCC25922	40 ± 0.64	35 ± 85	32 ± 0.75	23 ± 0.95
*P. aeruginosa* (ATCC27853)	42 ± 0.85	30 ± 0.75	28 ± 0.56	12 ± 0.64
*K. pneumonia* RCMB005 001 “2”	50 ± 0.98	50 ± 68	49 ± 0.79	16 ± 0.53
Fungi				Ketoconazole
*C. albicans* ATCC 10231	18 ± 0.83	17 ± 0.59	17 ± 0.88	20 ± 0.26
*C. tropicalis* RCMB005 004	15 ± 0.34	12 ± 0.76	12 ± 0.96	18 ± 0.49
*F. oxysporium* RCMB008 001 “2”	NA	NA	NA	26 ± 0.55
*A. flavus* RCMB 002002	NA	NA	NA	16 ± 0.36
NA	No activity			

## Data Availability

Data is contained within the article.
